# Pomegranate-Derived Polyphenols Reduce Reactive Oxygen Species Production via SIRT3-Mediated SOD2 Activation

**DOI:** 10.1155/2016/2927131

**Published:** 2016-10-20

**Authors:** Chong Zhao, Takenori Sakaguchi, Kosuke Fujita, Hideyuki Ito, Norihisa Nishida, Akifumi Nagatomo, Yukimasa Tanaka-Azuma, Yoshinori Katakura

**Affiliations:** ^1^Graduate School of Systems Life Sciences, Kyushu University, 6-10-1 Hakozaki, Higashi-ku, Fukuoka 812-8581, Japan; ^2^Graduate School of Bioscience & Biotechnology, Kyushu University, 6-10-1 Hakozaki, Higashi-ku, Fukuoka 812-8581, Japan; ^3^Faculty of Health and Welfare Science, Okayama Prefectural University, 111 Kuboki, Soja, Okayama 719-1197, Japan; ^4^Morishita Jintan Co., Ltd., 2-11-1 Tsudayamate, Hirakata, Osaka 573-0128, Japan; ^5^Faculty of Agriculture, Kyushu University, 6-10-1 Hakozaki, Higashi-ku, Fukuoka 812-8581, Japan

## Abstract

Pomegranate-derived polyphenols are expected to prevent life-style related diseases. In this study, we evaluated the ability of 8 pomegranate-derived polyphenols, along with other polyphenols, to augment* SIRT3*, a mammalian* SIR2* homolog localized in mitochondria. We established a system for screening foods/food ingredients that augment the* SIRT3* promoter in Caco-2 cells and identified 3 SIRT3-augmenting pomegranate-derived polyphenols (eucalbanin B, pomegraniin A, and eucarpanin T_1_). Among them, pomegraniin A activated superoxide dismutase 2 (SOD2) through SIRT3-mediated deacetylation, thereby reducing intracellular reactive oxygen species. The other SIRT3-augmenting polyphenols tested also activated SOD2, suggesting antioxidant activity. Our findings clarify the underlying mechanisms involved in the antioxidant activity of pomegraniin A.

## 1. Introduction

Calorie restriction (CR) is the only experimental manipulation that is known to reduce the incidence of age-related disorders such as diabetes, cancer, and cardiovascular diseases in mammals [1]. Sirtuins are NAD^+^-dependent deacetylases that have been found to mediate the effects of CR and regulate lifespan in lower organisms [2]. In mammals, there are seven sirtuins (SIRT1–SIRT7) localized in diverse positions within cells [3]. SIRT3, a mammalian SIR2 homolog localized in the mitochondria, regulates several aspects of mitochondrial function, including metabolism, energy homeostasis, and modulation of the response to oxidative stress [4]. Mitochondrial free radical theory of ageing suggests that accumulated oxidative stress caused by reactive oxygen species (ROS) is a major factor in determining lifespan [5]. Recently, increasing attention has been paid to the anti-aging effects of SIRT3, including a reduction in oxidative stress by targeting superoxide dismutase 2 (SOD2) enzyme [6] and preventing age-related hearing loss by activating mitochondrial isocitrate dehydrogenase 2 (IDH2) [7]. Consequently, SIRT3-activating compounds are promising candidates for suppressing oxidative stress and reducing age-related disorders.

The pomegranate,* Punica granatum* L., is known to contain many kinds of ellagitannins and anthocyanins [8, 9]. A large body of* in vitro *and* in vivo* evidence indicates that pomegranate peel extracts possess antioxidant, anticarcinogenic, and anti-inflammatory properties that may be useful in the treatment and prevention of cancer, cardiovascular disease, diabetes, dental conditions, bacterial infections, and ultraviolet-induced skin damage [10–20]. Furthermore,* in vivo* experiments have shown that pomegranate juice exhibits an inhibitory effect toward age-related diseases such as Alzheimer's disease [21] and Parkinson's disease [22]. In the present study, we focused on the functionalities of pomegranate-derived polyphenols through augmentation of SIRT3. Pomegranate-derived polyphenols are difficult to be absorbed in intestine; thus in the present study, we investigated the effects of these polyphenols on intestinal cells by using human colorectal cancer cell line, Caco-2, as a model cell [23].

## 2. Materials and Methods

### 2.1. Cell Culture and Reagents

The Caco-2 human colorectal cancer cell line (Riken Bioresource Center, Tsukuba, Japan) was cultured in Dulbecco's modified Eagle's medium (DMEM; Nissui, Tokyo, Japan) supplemented with 10% heat-inactivated fetal bovine serum (Life Technologies, Gaithersburg, MD, USA) at 37°C in an atmosphere containing 5% CO_2_.

### 2.2. Polyphenols

Polyphenols used in this study are listed in Table 1. The structure of ellagitannins is described elsewhere [9].

### 2.3. Screening System for Foods Augmenting the* SIRT3* Promoter

We amplified the human* SIRT3* promoter (−653 to −1) by PCR using human genomic DNA as a template, which was then cloned into the promoter-less Enhanced Green Fluorescent Protein- (EGFP-) expressing vector (pEGFP-C3, Takara, Shiga, Japan) and designated pSIRT3p-EGFP. Caco-2 cells transduced with pSIRT3p-EGFP were used to evaluate human* SIRT3* promoter activity. Changes in the EGFP fluorescence derived from pSIRT3p-EGFP were monitored by an IN Cell Analyzer 1000 (GE Healthcare, Amersham Place, UK).

### 2.4. Quantitative Reverse Transcription Polymerase Chain Reaction

RNA was extracted using the High Pure RNA Isolation Kit (Roche, Tokyo, Japan), and cDNA was prepared as previously described [24]. Quantitative reverse transcription polymerase chain reaction (qRT-PCR) was performed using Thunderbird SYBR qPCR Mix (Toyobo, Osaka, Japan) and a Thermal Cycler Dice Real Time System TP-800 instrument (Takara, Shiga, Japan) as previously described. Samples were analyzed in triplicate, and* SIRT3* expression levels were normalized to the corresponding* ACTB* (*β*-actin) levels. The PCR primer sequences used were as follows:* SIRT3 *forward primer 5′-CTGTACAGCAACCTCCAGCA-3′ and reverse primer 5′-CTCCTTGGCCAAAGTGAAAA-3′;* ACTB* forward primer 5′-TGGCACCCAGCACAATGAA-3′ and reverse primer 5′-CTAAGTCATAGTCCGCCTAGAAGCA-3′.

### 2.5. Assays for Antioxidant Defense System

SOD2 activity was measured with the SOD Assay Kit-WST (Dojindo, Kumamoto, Japan), following the manufacturer's instructions. The amount of reduced glutathione (GSH) and oxidized glutathione (GSSG) was measured with the GSSG/GSH Quantification Kit (Dojindo), following the manufacturer's instructions.

### 2.6. Immunoprecipitation

SOD2 cDNA fragments derived from pBI-EGFP-MnSOD (#16612; Addgene, Cambridge, MA, USA) were cloned into the FLAG-pcDNA3 vector and designated as pcDNA3-Flag-SOD2. Caco-2 cells were transduced with pcDNA3-Flag-SOD2 and treated with polyphenols (10 *μ*M) for 48 h. Proteins were extracted by NP-40 lysis buffer (0.5% NP-40, 5 mM ethylenediaminetetraacetic acid (EDTA), 2 mM Na_3_VO_4_, 10 mM Tris-Cl pH 7.6, 150 mM NaCl, 5 *μ*g/mL aprotinin, and 1 mM phenylmethylsulfonyl fluoride (PMSF)). SOD2 was immunoprecipitated with anti-Flag antibodies (M2; Sigma, St. Louis, MO, USA) and then immobilized with PureProteome Protein G Magnetic Beads (Merck Millipore, Billerica, MA, USA) overnight at 4°C. Immunoprecipitates were eluted with sample buffer (1 M Tris-HCl pH 6.8, 10% SDS, and 10% 2 ME).

### 2.7. Immunoblotting

Cell lysates were resolved by electrophoresis using 12% SDS-PAGE and transferred to a Hybond P membrane (GE Healthcare). The membrane was probed with an anti-Flag M2 antibody, anti-acetylated-lysine antibody (Cell Signaling Technology, Danvers, MA, USA), or anti-*β*-actin antibody (G043; Applied Biological Materials Inc., Richmond, BC, Canada). Horseradish peroxidase-labeled anti-rabbit IgG antibody (GE Healthcare) and anti-mouse IgG antibody (GE Healthcare) were used as secondary antibodies. The proteins were detected using ImmunoStar LD (Wako Pure Chemical, Osaka, Japan) and visualized with a LAS-1000 Lumino Image analyzer (Fuji Film, Tokyo, Japan).

### 2.8. Retrovirus Production and Transduction

Viral supernatants were produced after transfecting 293 T cells with pGag-pol, pVSV-G, and individual expression vectors (pSUPER-puro, pSUPER-puro-shSIRT1, and pSUPER-puro-shSIRT3) using the HilyMax reagent (Dojindo) as previously described [25]. The cells were cultured at 37°C in DMEM supplemented with 10% FBS for 24 h. Medium was replaced with fresh DMEM supplemented with 2% FBS and incubated for an additional 24 h. Viral supernatant was collected and supplemented with 10 mg/mL polybrene (Merck Millipore). The target cells were infected with this viral supernatant for 24 h at 37°C. After infection, the cells were selected with 3 *μ*g/mL puromycin (Enzo Life Sciences, Farmingdale, NY) for 3 days.

### 2.9. Short Hairpin RNA

The oligonucleotides containing siRNA-expressing sequences targeting human* SIRT1* and* SIRT3* were annealed (shSIRT1 top: 5′- GATCCCCGCAAAGCCTTTCTGAATCTATTTCGAAGAGATAGATTCAGAAAGGCTTTGCTTTTTA-3′, shSIRT1 bottom: AGCTTAAAAAGCAAAGCCTTTCTGAATCTATCTCTTCGAAATAGATTCAGAAAGGCTTTGCGGG-3′; shSIRT3 top: 5′-GATCCCCCCAACGTCACTCACTACTTTTTCGAAGAGAAAGTAGTGAGTGACGTTGGGTTTTTA-3′, shSIRT3 bottom: 5′-AGCTTAAAAACCCAACGTCACTCACTACTTTCTCTTCGAAAAAGTAGTGAGTGACGTTGGGGGG-3′) and cloned into the pSUPER.retro.puro (OligoEngine, Seattle, WA, USA).

### 2.10. Evaluation of ROS Generation

Production of intracellular ROS in Caco-2 cells was measured using BES-H_2_O_2_-Ac (Wako Pure Chemical) according to the manufacturer's protocol. Caco-2 cells were treated with 10 *μ*M pomegraniin A for 48 h or 10 mM N-acetyl cysteine (NAC) for 2 h before analyzing ROS production. The medium was removed and cells were washed twice with 4-(2-hydroxyethyl)-1-piperazineethanesulfonic acid (HEPES) buffer (pH 7.4). The cells were incubated with 5 *μ*M BES-H_2_O_2_-Ac and Hoechst 33342 (Dojin) in HEPES (pH 7.4) at 37°C for 30 min, followed by detection with an IN Cell Analyzer 1000 (GE Healthcare).

### 2.11. Statistical Analysis

All experiments were performed at least 3 times, and the corresponding data are shown. The results are presented as mean ± standard deviation. Statistical significance was determined by one-way ANOVA with Tukey's* post hoc* test. Statistical significance was defined as *P* < 0.05 (^*∗*^ *P* < 0.05; ^*∗∗*^ *P* < 0.01).

## 3. Results

### 3.1. Identification of Polyphenols That Augment* SIRT3* Transcription in Caco-2 Cells

**Figure 1:**
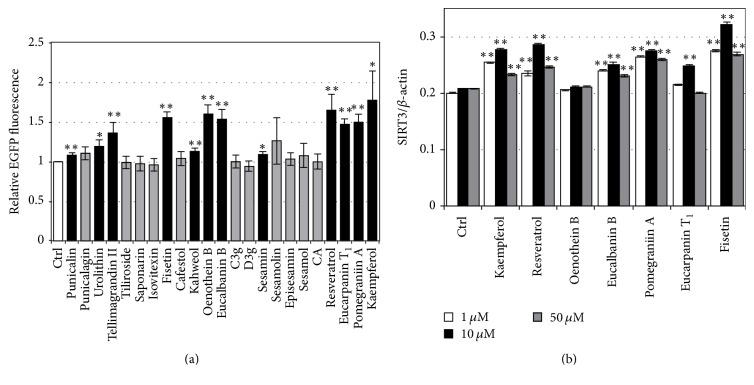
Identification of polyphenols that augment* SIRT3* transcription in Caco-2 cells. Polyphenols (10 *μ*M) and their corresponding amount of DMSO were added to Caco-2 cells transduced with SIRT3p-EGFP and changes in EGFP fluorescence were monitored by an IN Cell Analyzer 1000 (a). Dose-dependent effects of polyphenols on the expression of endogenous* SIRT3* in Caco-2 cells were assessed by qRT-PCR. Control Caco-2 cells were treated with the same volume of DMSO used for treating with 1, 10, and 50 *μ*M of polyphenols. (b) The results are expressed as mean ± standard deviation. Statistical significance between the respective concentration and its corresponding in the control was determined by one-way ANOVA with Tukey's* post hoc* test. Statistical significance was defined as *P* < 0.05 (^*∗*^ *P* < 0.05;  ^*∗∗*^ *P* < 0.01). C3g, cyanidin 3-glucoside chloride; D3g, delphinidin 3-glucoside chloride; CA, chlorogenic acid.

Previously, we established a system for screening foods/food ingredients that augment the* SIRT1* promoter in Caco-2 cells and identified* Lactobacillus brevis* T2102 as* SIRT1*-activating lactic acid bacterium [26]. Here, we established a similar system for screening foods/food ingredients that augment the* SIRT3* promoter in Caco-2 cells, where pSIRT3p-EGFP was transduced. We tested 11 polyphenols, 5 flavonoids, 2 terpenoids, and 4 lignans in this assay. As shown in Figure 1(a), several samples including tellimagrandin II, fisetin, oenothein B, eucalbanin B, resveratrol, eucarpanin T_1_, pomegraniin A, and kaempferol strongly augmented* SIRT3* promoter activity in Caco-2 cells. In addition, we tested whether these polyphenols augment endogenous* SIRT3* expression in Caco-2 cells by qRT-PCR. Dose-dependent change in polyphenol-induced SIRT3 augmentation showed that treatment with 10 *μ*M polyphenols is most effective and that polyphenols, including eucalbanin B, pomegraniin A, eucarpanin T_1_, and fisetin, increased endogenous* SIRT3* expression in Caco-2 cells to a level comparable to that observed in the positive control samples treated with kaempferol and resveratrol (Figure 1(b)).

### 3.2. Effects of* SIRT3*-Augmenting Polyphenols on SOD2 Activity

**Figure 2:**
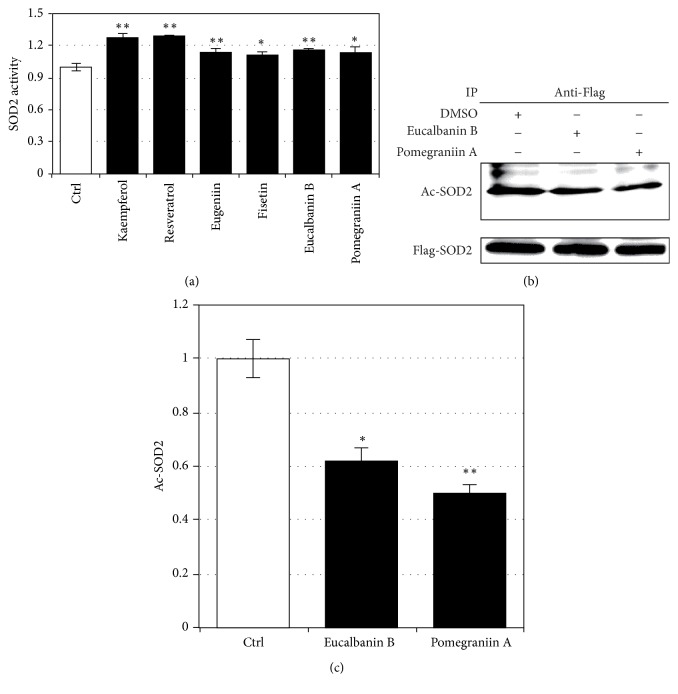
Effects of* SIRT3*-augmenting polyphenols on SOD2 activity. Caco-2 cells were treated with SIRT3-augmenting polyphenols (10 *μ*M) for 48 h and SOD2 activity was measured using the SOD Assay Kit-WST (a). Deacetylation of SOD2 by pomegraniin A; Caco-2 cells were transduced with Flag-SOD2 and treated with 10 *μ*M of eucalbanin B or pomegraniin A for 48 h. Flag-SOD2 was immunoprecipitated with anti-Flag M2 antibodies immobilized to Protein G and analyzed by immunoblot using anti-acetylated-lysine antibodies (b and c). Band intensity corresponding to acetylated SOD2 was quantitatively determined using Image J software. The results are expressed as mean ± standard deviation. Control Caco-2 cells were treated with the same volume of DMSO used for treating with 10 *μ*M of polyphenols. Statistical significance between the respective polyphenol and control was determined by one-way ANOVA with Tukey's* post hoc* test. Statistical significance was defined as *P* < 0.05 (^*∗*^ *P* < 0.05; ^*∗∗*^ *P* < 0.01).

Qiu et al. reported that in 293 T cells SIRT3 activates SOD2, a major mitochondrial antioxidant enzyme [6]; therefore, we examined whether SIRT3-augmenting polyphenols activate SOD2 activity in Caco-2 cells. Our results show that all polyphenols tested activate SOD2 activity in Caco-2 cells (Figure 2(a)), and foods/food ingredients that activate SOD2 activity can be screened by our system, which targets the* SIRT3* promoter. Among these polyphenols, eucalbanin B and pomegraniin A are polyphenols that have not been reported as a sirtuin activator and reproducibly activate SOD2 as well as augmenting SIRT3 expression in independent experiment; thus we used these polyphenols for further experiments.

Qiu et al. reported that SIRT3 deacetylates and activates SOD2 [6]; therefore, we wanted to clarify the molecular mechanisms for activation of SOD2 by SIRT3-augmenting polyphenols. We tested whether SIRT3-augmenting polyphenols deacetylate SOD2 in Caco-2 cells, with a focus on the activity of eucalbanin B and pomegraniin A, which are derived from pomegranate [9]. Caco-2 cells were transfected with Flag-SOD2 and treated with the test polyphenols; SOD2 was then immunoprecipitated with anti-Flag antibodies. The acetylation status of immunoprecipitated SOD2 was evaluated by immunoblotting using anti-acetylated-lysine antibodies (Figure 2(b)). Results show that eucalbanin B and pomegraniin A significantly attenuated acetylation of SOD2 (Figures 2(b) and 2(c)), suggesting that these SIRT3-augmenting polyphenols activate SOD2 by deacetylation.

### 3.3. Pomegraniin A Activation of SOD2 Is Dependent on SIRT3

**Figure 3:**
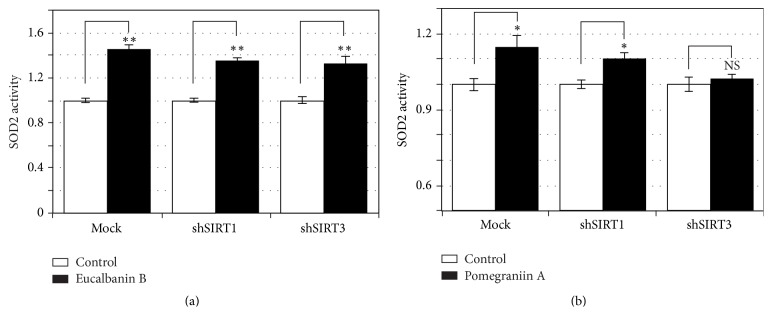
SIRT3-dependency of pomegraniin A-induced SOD2 activation. Effects of eucalbanin B (a) and pomegraniin A (b) on SOD2 activity in* SIRT1*- and* SIRT3*-silenced Caco-2 cells were evaluated using the SOD Assay Kit-WST. The results are expressed as mean ± standard deviation. Control Caco-2 cells were treated with the same volume of DMSO used for treating with 10 *μ*M of polyphenols. Statistical significance between the respective polyphenol and control was determined by one-way ANOVA with Tukey's* post hoc* test. Statistical significance was defined as *P* < 0.05 (^*∗*^ *P* < 0.05; ^*∗∗*^ *P* < 0.01). SOD2, superoxide dismutase 2.

To further clarify the involvement of SIRT3 in the activation of SOD2 by eucalbanin B and pomegraniin A, we examined their effects on SOD2 activity in* SIRT1*- or* SIRT3*-silenced Caco-2 cells (Figure 3). Results show that eucalbanin B and pomegraniin A did not attenuate SOD2 activation in* SIRT1*-silenced Caco-2 cells. Furthermore, activation of SOD2 by pomegraniin A, but not by eucalbanin B, was significantly reduced in* SIRT3*-silenced Caco-2 cells, suggesting that pomegraniin A activates SOD2 in a SIRT-3 dependent manner. However, molecular mechanisms for eucalbanin B-induced activation of SOD2 remain unclear.

### 3.4. Pomegraniin A Reduced ROS Levels in Caco-2 Cells

**Figure 4:**
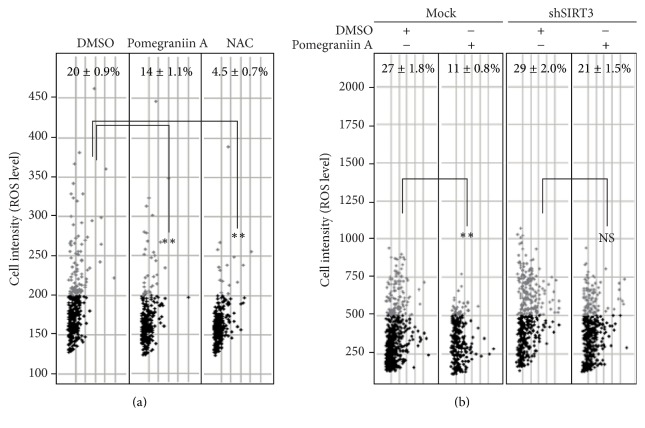
Pomegraniin A reduces intracellular ROS levels via SIRT3. Effects of pomegraniin A on intracellular ROS levels in Caco-2 cells; cells were treated with 10 *μ*M pomegraniin A for 48 h or 10 mM NAC for 2 h prior to detection, and intracellular ROS levels were analyzed with an IN Cell Analyzer 1000. The relative number of the cells with high ROS levels was shown in the scatter plot (a). Effects of pomegraniin A on intracellular ROS levels in* SIRT3*-silenced Caco-2; cells were treated with 10 *μ*M pomegraniin A for 48 h, followed by detection of ROS levels with an IN Cell Analyzer 1000. The relative number of the cells with high ROS levels was shown in the scatter plot (b). Control Caco-2 cells were treated with the same volume of DMSO used for treating with 10 *μ*M of polyphenols. Statistical significance between samples and control was determined by one-way ANOVA with Tukey's* post hoc* test. Statistical significance was defined as *P* < 0.05  (^*∗∗*^ *P* < 0.01). NAC, N-acetyl cysteine; ROS, reactive oxygen species; DMSO, dimethyl sulfoxide. Experiments were repeated three times, and representative was shown.

As described above, pomegraniin A activates SOD2 in Caco-2 cells, suggesting that it has antioxidant properties. Therefore, we investigated the effects of pomegraniin A on intracellular ROS levels using an IN Cell Analyzer 1000 that allowed collection of fluorescent images derived from BES-H_2_O_2_. We evaluated intracellular ROS levels by analyzing cell size (*x*-axis) as well as the percentage of BES-H_2_O_2_-positive/negative cells (*y*-axis). The scatter plot shows that the relative number of cells with high ROS levels decreased upon treatment with pomegraniin A and NAC. Specifically, the relative number of cells with high ROS levels decreased from 20% (control) to 14% and 4.5% by the treatment with pomegraniin A and NAC, respectively (Figure 4(a)). Furthermore, the reduction in ROS levels observed in cells treated with pomegraniin A was attenuated in* SIRT3*-silenced Caco-2 cells (Figure 4(b)). These results indicate that pomegraniin A-associated decreases in intracellular ROS levels are dependent on SIRT3 expression.

### 3.5. Effects of* SIRT3*-Augmenting Polyphenols on Glutathione Antioxidant System

**Figure 5:**
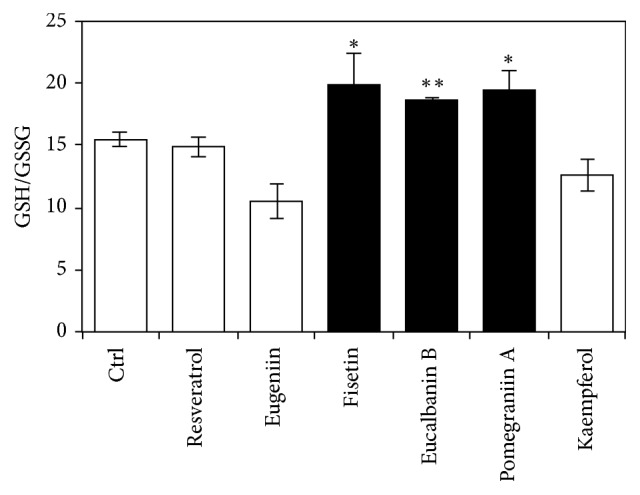
Effects of SIRT3-augmenting polyphenols on glutathione antioxidant system. Caco-2 cells were treated with SIRT3-augmenting polyphenols (10 *μ*M) for 48 h, and the amount of reduced glutathione (GSH) and oxidized glutathione (GSSG) was measured with the GSSG/GSH Quantification Kit, following the manufacturer's instructions. Control Caco-2 cells were treated with the same volume of DMSO used for treating with 10 *μ*M of polyphenols. Statistical significance between the respective polyphenol and control was determined by one-way ANOVA with Tukey's* post hoc* test. Statistical significance was defined as *P* < 0.05 (^*∗*^ *P* < 0.05; ^*∗∗*^ *P* < 0.01).

Someya et al. reported that SIRT3 increased ratio of reduced-to-oxidized glutathione through direct deacetylation of mitochondrial isocitrate dehydrogenase 2 [7]; then we examined whether SIRT3-augmenting polyphenols activate glutathione antioxidant system as well as SOD2 in Caco-2 cells. Results show that 3 polyphenols among SIRT3-augmenting polyphenols increased the ratio of GSH/GSSG, although all polyphenols activate SOD2 activity (Figure 5).

## 4. Discussion

Antioxidant activity and* in vivo* functionalities of pomegranate extract such as repair and maintenance of hepatic and intestinal function and attenuation of inflammation have been extensively studied [13–20]. Ellagitannins, anthocyanidins, and related polyphenols are thought to be efficient antioxidants extracted from pomegranate (*Punica granatum* L.). Previous studies have shown that three major anthocyanidins (delphinidin, cyanidin, and pelargonidin) contribute to the antioxidant activity of pomegranate fruit [27]. Recently, studies have shown that new ellagitannin oligomers isolated from pomegranate exhibit potent inhibitory effects on the formation of advanced glycation end-products (AGEs), which have been associated with life-style related diseases including Alzheimer's disease [9]. Therefore, a better understanding of the mechanisms underlying the antioxidant activity of pomegranate-derived polyphenols is critical for their further development as anti-ageing chemopreventive agents. For this purpose, we report here that pomegraniin A has potent antioxidant properties that are dependent on SIRT3-mediated SOD2 activation in Caco-2 cells.

Several food-derived bioactive polyphenols have been reported to attenuate cellular oxidative stress, and thus it has been suggested that they have the potential to prevent age-related diseases [28, 29]. These polyphenols exert their antioxidant activity through activation of various signaling pathways, including JAK-STAK, MAPK/ERK, NF-*κ*B, and MAPK p38/JNK [30]. Several polyphenols (resveratrol, black chokeberry (*Aronia melanocarpa*) extract, and green tea polyphenols) have been shown to activate SIRT3 [31–33]. In particular, decreased SIRT3 expression and increased SOD2 acetylation resulting from a high-fat diet in Wister rats were attenuated by administration of green tea polyphenols, which likely explains the reduction in oxidative stress observed in the kidney tissues of the rats [32]. In the present study, we clarified the SIRT3-augmenting activity of the polyphenol pomegraniin A, which activates SOD2 and reduces intracellular ROS in an* in vitro* system. These data provide direct evidence for the functionality of SIRT3-augmenting polyphenols [33]. Furthermore, these results confirm the utility of the model described here for identifying foods/food ingredients that increase SIRT3 expression and consequently reduce intracellular ROS. Although eucalbanin B was also identified as a SIRT3-augmenting polyphenol in this system, SOD2 activity increased in eucalbanin B-treated cells was not attenuated in the* SIRT3*-silenced Caco-2 cells. These results suggest that eucalbanin B activates SOD2 via a pathway that is distinct from SIRT3 activation, which will be pursued further in a future study.

In the present study, we identified 6 polyphenols among 22 polyphenols that augment SIRT3 expression and activate SOD2. Although we focused on eucalbanin B and pomegraniin A as SIRT3-augmenting polyphenols, other polyphenols may elicit their effects by mechanisms similar to pomegraniin A or by activating individual pathways. Until now, a limited number of polyphenols and food derived extracts were known to activate SIRT3 and showed distinct functions. Black chokeberry extract regulates lipid metabolism and lipoprotein assembly through the activation of SIRT3 [31]. NAD^+^ and grape wine polyphenols prevent axonal apoptosis through SIRT3-dependent mechanisms [34]. Resveratrol activates SIRT3, which then ameliorates cardiac fibrosis and improves cardiac function via the transforming growth factor-*β*/Smad3 pathway [35]. Thus, other SIRT3-augmenting polyphenols have distinct functions that must be clarified in future studies.

Although pomegraniin A activation of SOD2 occurs only via SIRT3, several polyphenols, such as kaempferol, have been reported to activate both SIRT1 and SIRT3 [36]. Furthermore, activation of SIRT1 is known to promote the nuclear factor-E2 related factor 2- (Nrf2-) antioxidant response element (ARE) pathway [37]. Here, we determined that the antioxidant activity of pomegraniin A occurs through activation of the* SIRT3*-*SOD2* pathway. Although pomegraniin A and eucalbanin B are ellagitannin tetramer and dimer, respectively [9], with similar structural features, SOD2 activation by these ellagitannins showed distinct dependency on SIRT3. SOD2 activation by pomegraniin A highly depended on SIRT3, but that by eucalbanin B did not, suggesting that eucalbanin B activates SOD2 by not only SIRT3, but also other molecules such as SIRT1 as described previously [38]. In future studies, we will clarify additional mechanisms associated with the antioxidant activity of pomegraniin A.

Superoxide is one of the most abundant ROS produced by mitochondria. The extent of ROS-induced cellular damage accumulates with age. Thus, SOD-catalyzed reductions in ROS levels function to regulate intracellular ROS levels. In particular, overexpression of SOD in model organisms, including* Saccharomyces cerevisiae* and* Drosophila*, reduces oxidative damage and extends lifespan [39]. Currently, the potential for pomegranate-derived polyphenols to prevent and treat life-style related diseases has been extensively reported [10–12]. SOD2 activation by pomegraniin A likely provides a molecular basis for the broad spectrum of antioxidant activity observed for pomegranate juice. However, polyphenols having strong antioxidant activities do not always show similar biological activities* in vivo* due to their bioavailability, uptake, and metabolism. Thus, we must clarify biological activity of pomegraniin A* in vivo* as well as its intestinal uptake, metabolism, and kinetics in blood by using animal models and reveal the molecular basis of* in vivo* function of pomegraniin A.

SOD2 and isocitrate dehydrogenase 2, which regulates the ratio of GSH to GSSG, were reported to be targets of SIRT3 [40]; thus we investigated the effects of these polyphenols on SOD2 activity and GSH/GSSG ratio. Among SIRT3-augmenting polyphenols, 3 polyphenols including fisetin, eucalbanin B, and pomegraniin A activated both SOD2 and glutathione antioxidant system, while other polyphenols including resveratrol, eugeniin, and kaempferol only activated SOD2. These results suggest that there exist polyphenol-dependent activation mechanisms for cellular antioxidant system. Furthermore, we would investigate the effects of these polyphenols on other antioxidant enzymes including SOD1, glutathione peroxidase, glutathione reductase, and catalase in the future study.

In summary, we identified a pomegranate-derived polyphenol, pomegraniin A, that eliminates ROS through SIRT3-dependent SOD2 activation in Caco-2 cells, which would ameliorate intestinal injury induced by hemorrhagic shock as reported previously [41]. Furthermore, this effect of pomegraniin A on intestinal cells might lead to the activation of interorgan interaction and prevention of age-related disorders, the possibility of which must be evaluated in the future study [23]. However, the homogenate of fresh arils (2 kg) of pomegranate contains only 11 mg (3.5 *μ*mol) of pomegraniin A. Thus, daily intake of pomegraniin A as a supplement is thought to be more desirable.

## Figures and Tables

**Table 1:** Polyphenols.

Classification	Name	Origin
Polyphenol	Punicalin	Pomegranate
	Punicalagin	Pomegranate
	Oenothein B	Pomegranate
	Eucalbanin B	Pomegranate
	Eucarpanin T_1_	Pomegranate
	Pomegraniin A	Pomegranate
	Urolithin A	Ellagitannin metabolite
	Chlorogenic acid	Coffee
	Resveratrol	Grape
	Tellimagrandin II	Rose

Anthocyanin	Cyanidin 3-glucoside chloride	Pomegranate
	Delphinidin 3-glucoside chloride	Pomegranate

Flavonoid	Tiliroside	Rose hip
	Saponarin	Green barley
	Isovitexin	Green barley
	Fisetin	Green barley
	Kaempferol	Tea

Terpenoid	Cafestol	Coffee beans
	Kahweol	Coffee beans

Lignan	Sesamin	Sesame
	Sesamolin	Sesame
	Episesamin	Sesame
	Sesamol	Sesame
